# Assessment of Myocardial Function and Injury by Echocardiography and Cardiac Biomarkers in African Children With Severe *Plasmodium falciparum* Malaria*

**DOI:** 10.1097/PCC.0000000000001411

**Published:** 2018-03-02

**Authors:** Simon Kotlyar, Peter Olupot-Olupot, Julius Nteziyaremye, Samuel O. Akech, Sophie Uyoga, Rita Muhindo, Christopher L. Moore, Kathryn Maitland

**Affiliations:** 1 Department of Emergency Medicine, Yale School of Medicine, New Haven, CT.; 2 London School of Hygiene and Tropical Medicine, London, United Kingdom.; 3 Mbale Clinical Research Institute, Mbale Regional Referral Hospital, Mbale, Uganda.; 4 Kenya Medical Research Institute (KEMRI)-Wellcome Trust Research Programme, Kilifi, Kenya.; 5 Department of Pediatrics, Faculty of Medicine, Wellcome Trust Centre for Global Health, Imperial College, London, United Kingdom.

**Keywords:** cardiac, echocardiography, falciparum, malaria, pediatric

## Abstract

**Objectives:**

Perturbed hemodynamic function complicates severe malaria. The Fluid Expansion as Supportive Therapy trial demonstrated that fluid resuscitation, involving children with severe malaria, was associated with increased mortality, primarily due to cardiovascular collapse, suggesting that myocardial dysfunction may have a role. The aim of this study was to characterize cardiac function in children with severe malaria.

**Design:**

A prospective observational study with clinical, laboratory, and echocardiographic data collected at presentation (T0) and 24 hours (T1) in children with severe malaria. Cardiac index and ejection fraction were calculated at T0 and T1. Cardiac troponin I and brain natriuretic peptide were measured at T0. We compared clinical and echocardiographic variables in children with and without severe malarial anemia (hemoglobin < 5 mg/dL) at T0 and T1.

**Setting:**

Mbale Regional Referral Hospital.

**Patients:**

Children 3 months to 12 years old with severe *falciparum* malaria.

**Interventions:**

Usual care.

**Measurements and Main Results:**

We enrolled 104 children, median age 23.3 months, including 61 children with severe malarial anemia. Cardiac troponin I levels were elevated (> 0.1 ng/mL) in *n* equals to 50, (48%), and median brain natriuretic peptide was within normal range (69.1 pg/mL; interquartile range, 48.4–90.8). At T0, median Cardiac index was significantly higher in the severe malarial anemia versus nonsevere malarial anemia group (6.89 vs 5.28 L/min/m^2^) (*p* = 0.001), which normalized in both groups at T1 (5.60 vs 5.13 L/min/m^2^) (*p* = 0.452). Cardiac index negatively correlated with hemoglobin, *r* equals to –0.380 (*p* < 0.001). Four patients (3.8%) had evidence of depressed cardiac systolic function (ejection fraction < 45%). Overall, six children died, none developed pulmonary edema, biventricular failure, or required diuretic treatment.

**Conclusions:**

Elevation of cardiac index, due to increased stroke volume, in severe malaria is a physiologic response to circulatory compromise and correlates with anemia. Following whole blood transfusion and antimalarial therapy, cardiac index in severe malarial anemia returns to normal. The majority (> 96%) of children with severe malaria have preserved myocardial systolic function. Although there is evidence for myocardial injury (elevated cardiac troponin I), this does not correlate with cardiac dysfunction.

## Abstract 2

Supplemental Digital Content is available in the text.

Impaired hemodynamic function is a recognized complication of severe *Plasmodium falciparum* malaria (1). In African children with severe malaria (SM), clinical markers of impaired perfusion are frequent and associated with increased mortality (2–8). While parasite sequestration is the hallmark of malaria pathogenesis, at a clinical level, the production of inflammatory cytokines and acidosis have strong synergies with bacterial sepsis (9, 10). Impaired cardiac function has long been established as a component of hypoperfusion secondary to septic shock; however, the role of cardiac systolic function in SM has not been well described (11–13).

The Fluid Expansion as Supportive Therapy trial demonstrated increased mortality in bolus arms compared with controls (no bolus) in African children with severe febrile illness (including a large subgroup of malaria) (14). There was no evidence to indicate that fluid overload was related to the increased mortality associated with fluid boluses. A subsequent analysis of the terminal clinical events (TCEs) showed that the major difference between bolus and control arms was a higher proportion of cardiogenic shock TCE in bolus arms (*n* = 123; 4.6% vs 2.6%; *p* = 0.008) and not due to respiratory or neurologic TCEs (15). What remains unclear is to what extent myocardial dysfunction accompanied the clinical presentation and compromised the response to fluid bolus therapy.

SM is frequently complicated by anemia requiring transfusion. An in-depth understanding of cardiac function in children with SM is essential to the development of management guidelines. The purpose of this study was to assess cardiac systolic function using echocardiography and laboratory markers of myocyte injury in children with severe *P. falciparum* malaria on a pediatric ward typical of a low-resource setting in Africa.

## MATERIALS AND METHODS

We conducted a prospective observational study of children presenting with severe *P. falciparum* malaria to Mbale Regional Referral Hospital (MRRH), Eastern Uganda, an area of high perennial malaria transmission.

### Population

Children 3 months to 12 years old presenting with presence or history of fever with signs or symptoms consistent with SM as defined by modified World Health Organization criteria (16) and a positive rapid diagnostic test (RDT) for *P. falciparum* malaria (OpitiMAL; Diamed, Fribourg, Switzerland) were eligible for the study. Informed consent was obtained from a parent or guardian. Case definition for “SM” included a malaria positive slide or RDT plus evidence of impaired consciousness, respiratory distress, or severe anemia (hemoglobin < 5 g/dL). Cerebral malaria was defined as unrousable coma (Glasgow Coma Score < 8 [age 5–12 yr] or Blantyre Coma Score < 3 [age 0–4 yr]) lasting more than 30 minutes after a seizure and absence of other causes of coma.

The study was approved by the MRRH Institutional Review Committee and the London School of Hygiene and Tropical Medicine Ethics Committee.

### Data Collection and Treatment

Demographic and clinical data were collected using standardized forms at admission. Nutritional status was assessed by height, weight, and mid-upper arm circumference (MUAC). Hemoglobin (g/dL) was determined by HemoCue (Angelholm, Sweden) at admission and 24 hours. Blood glucose (Acon Labs, San Diego CA) was determined at admission and every 8 hours until the child was conscious. Lactate was determined at admission (Lactate Pro; Arkray Labs, Amsterdam, The Netherlands). Cardiac troponin I (cTnI) and brain natriuretic peptide (BNP) were batch assayed from admission samples using enzyme-linked immunosorbent assay (ELH-CTNI and ELHBNP RayBio Elisa; RayBiotech, Norcoss, GA). All children received standard treatments in accordance to Uganda national guidelines (17). Children with severe anemia were transfused with 20 mL/Kg whole blood. Fluid administration was given at the discretion of the treating clinician.

### Echocardiography

A portable Phillips CX50 Ultrasound System (Philips Healthcare, Andover, MA) equipped with an S5-1 phased array cardiac probe (1–5 MHz) was used. Sonographic measurements were obtained at admission (T0) and 24 hours (T1) by the study author (S.K.). Measurements were recorded and processed using preset software. All images were digitally recorded in Digital Imaging and Communications in Medicine loops and still frames and stored for review by a second reviewer (C.L.M.).

Measurements for the estimation of left ventricular (LV) ejection fraction (EF) were made in the apical four-chamber view by obtaining three separate readings in consecutive cardiac cycles for LV end-diastolic diameter (LVEDD) and LV end-systolic diameter (LVESD). LV EF was calculated using the Simpson method: EF = ([LVEDD_3_ – LVESD_3_]/LVEDD_3_) %.

Parasternal long axis views were used to obtain LV outflow tract (LVOT) diameter at mid-systole. Pulsed wave Doppler interrogation of the aortic valve with the sample gate positioned at the valve leaflets was used to obtain a velocity time integral (VTI) at the LVOT. Measurements were made for three consecutive cardiac cycles and then averaged. Stroke volume (SV) was calculated using the equation: SV = VTI × cross-sectional area of the aorta, with (cross-sectional area of the aorta = 0.785 × aortic valve diameter^2^). Cardiac output (SV × heart rate [HR]) was then corrected for total body surface area (TBSA), and a cardiac index (CI) was calculated as: CI = cardiac output/TBSA.

### Analysis and Statistics

Data were entered on Filemaker Pro (Santa Clara, CA) and exported into the Statistical Package for the Social Science (SPSS v22, Chicago, IL) for analysis. TBSA was calculated using the methodology described by Mosteller (18). Significance testing for categorical variables was carried out using chi-square testing for large samples and Fisher exact for small sample sizes. Continuous variables were not normally distributed, and medians with interquartile range (IQR) are reported with nonparametric Mann-Whitney *U* testing for significance. Pearson correlation coefficients for CI and clinical metrics were calculated for bivariate linear correlation using two-tailed significance testing. Standardized values for cTnI (0.1 ng/mL) and BNP (100 pg/mL) in healthy controls were used as cutoff values for normal (19–27).

## RESULTS

A total of 104 children with SM were enrolled. Median age was 23.3 months (IQR, 8.6–33.1 mo), 57% were males. Summaries of baseline clinical and laboratory data are presented in **Table 1** overall and by subgroup SM anemia (SMA *n* = 61) and other SM (hemoglobin > 5 g/dL) (SM *n* = 43). Fifteen children (14.4%) were classified as having cerebral malaria, of which seven (46.7%) also had SMA. Significant clinical differences between the two subgroups were noted (Table 1). Impaired perfusion (systolic blood pressure < 75 mm Hg in children age 3–12 mo, < 85 mm Hg in children age 1–5 yr, and < 95 mm Hg in children age > 5 yr) was present in 19% of patients. Lactate was greater than or equal to 4 mmol/dL in 54% of children on presentation.

**TABLE 1.**
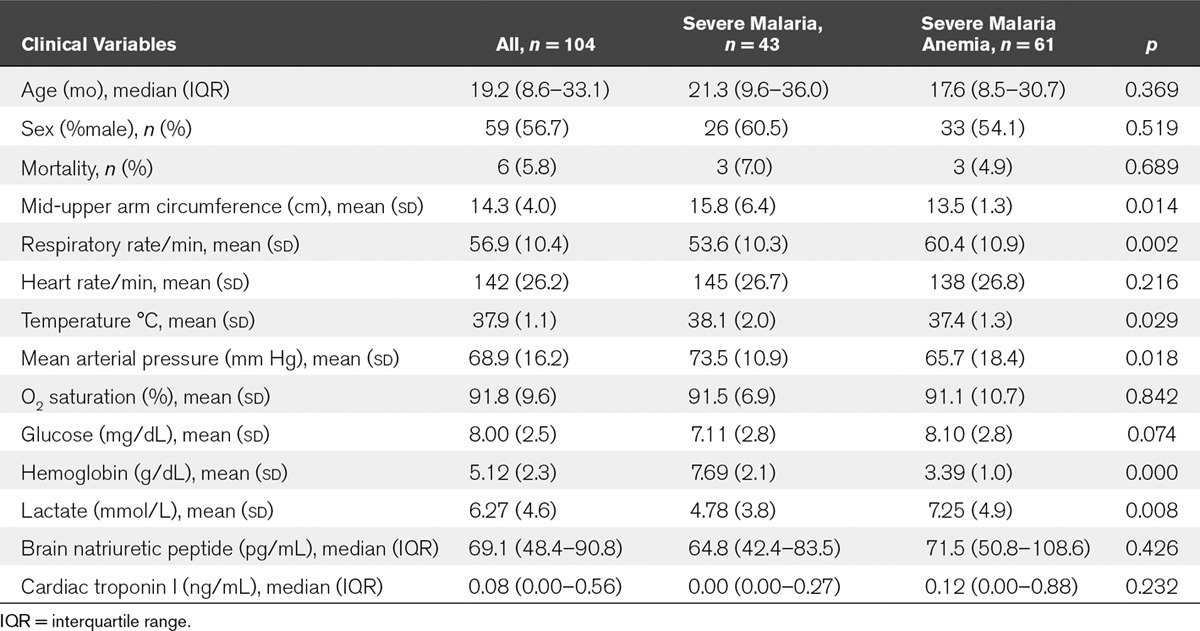
Demographic, Clinical, and Laboratory Baseline Data

### Echocardiographic Measurements

**Figure 1.**
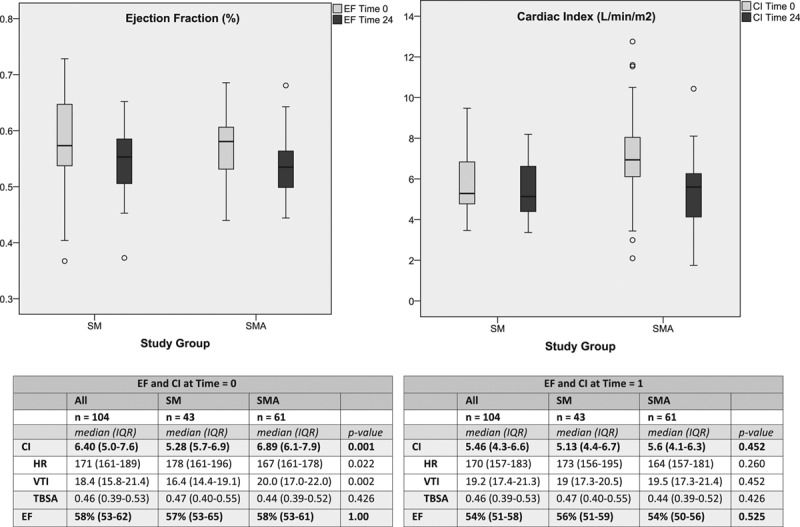
Ejection fraction (EF) (%) and cardiac index (CI) (L/min/m^2^) at time-0 (admission) and time-1 (24 hr). Median and interquartile range (IQR) for echocardiographic variables in severe malaria (SM) and severe malarial anemia (SMA) groups. HR = heart rate, TBSA = total body surface area (m^2^), VTI = velocity time integral.

Of the initial 104 patients who underwent echocardiography at T0, 93 (89.4%) were available for repeat assessment at T1. Of 11 patients who did not receive follow-up echocardiography, six died before 24 hours, four absconded, and one was discharged (recovered) prior to follow-up. Echocardiographic metrics at T0 and T1 are summarized in **Figure 1** with variables used to calculate CI and their summary statistics included for comparison.

### Myocardial Function at Admission and at 24 Hours

At T0, median CI was 6.4 L/min/m^2^ (IQR 5.0–7.6 L/min/m^2^) in the overall group and elevated above the reference range of 4.5 L/min/m^2^ in 87 of 104 (84%) of the cohort (28). CI was significantly higher in the SMA group than in the SM group at T0, 6.89 versus 5.28 L/m/m^2^ (*p* < 0.001). The primary component of this increase was an increased VTI (a surrogate of SV) in the SMA group, suggesting increased cardiac output. At T0, median EF was 58% (IQR, 53%–62%) and similar in the SM and SMA groups (*p* = 1.00). This admission EF was within normal range (that being greater than 45%) in 100 of 104 of participants (96.2%). Of the four with low EF, one child had severe anemia. One patient had EF equals to 31% with evidence of global hypokenisis (hemoglobin = 6.5) and expired shortly after arrival, another (EF 40%) had high levels of cTnI (7.35 ug/mL) and BNP (386 pg/mL) and had an uneventful recovery. The other two children (EF = 37% and 43%) recovered following resuscitation with normalization of cardiac function (EF = 54%, 56%).

Median CI in the SMA group significantly decreased (from 6.9 L/min/m^2^ [IQR, 6.1–7.9] at T0) to 5.6 L/min/m^2^ [IQR, 4.1–6.3] at T1 (*p* > 0.001). Median CI in the SM group remained constant at 5.3 L/min/m^2^ (IQR, 5.7–6.9) at T0 and 5.1 L/min/m^2^ (IQR, = 4.4–6.7) at T1 (*p* = 0.479).

### Fluid and Blood Transfusions Received

A total of 71 patients (68.3%) received either blood transfusion only (*n* = 61; 58.7%), crystalloid bolus only (*n* = 2; 1.9%), or both (*n* = 8; 7.7%). Of the 61 patients with SMA, 57 (93.4%) received blood transfusion on day 1. Of the four SMA patients who did not get blood transfusion, two died, one absconded, and one child survived receiving delayed transfusion at 72 hours when blood became available. In addition, 14 children (32.6%) with SM received blood transfusion due to a drop in hemoglobin following admission. Mean transfusion volume was 15.7 mL/kg (sd, 4.91) with no difference between groups (*p* = 0.987). Mean crystalloid volume over 24 hours was similar in both groups, 66.9 mL/kg (sd, 57.3) and 63.2 mL/kg (sd, 32.3) in the SM and SMA groups respectively (*p* = 0.914). No child received diuretics or inotropes. Clinically, we found no evidence of pulmonary edema during hospitalization. Overall six children died (5.8%), three in the SM and three in the SMA group. All six deaths occurred prior to 24-hour follow-up.

### Clinical Correlations With CI

**Figure 2.**
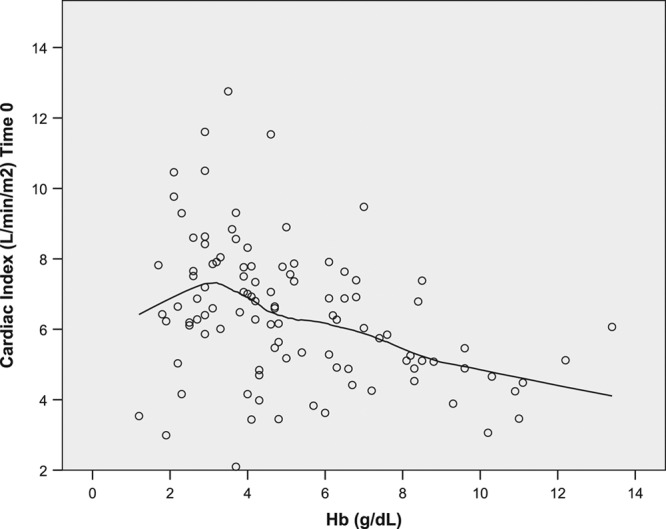
Scatter plot of cardiac index versus hemoglobin (Hb) at admission (T0). Nonparametric locally weighted scatter plot smoothing.

Pearson correlation coefficients for CI and key clinical and laboratory variables are presented in **Table 2**. The only significant correlation was with [hemoglobin]. This was a negative correlation, increase in CI for a decrease in [hemoglobin], with *r* equals to –0.380 (*p* < 0.001) **Figure 2**. We found no association between CI and lactate or mean arterial pressure (MAP).

**TABLE 2.**
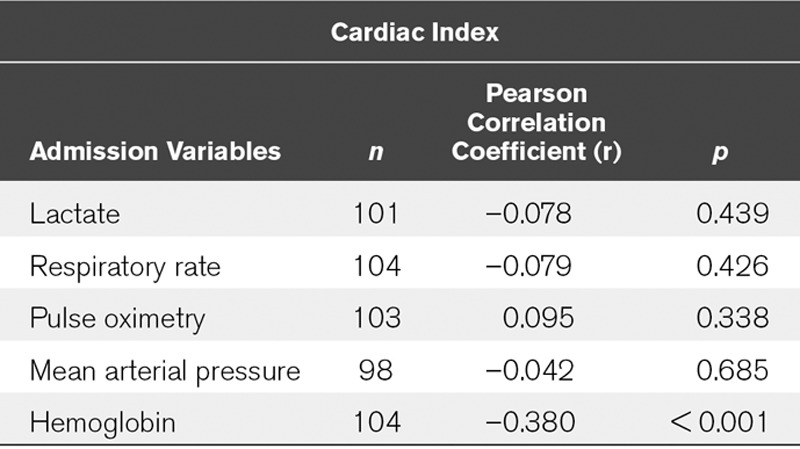
Pearson Correlation Coefficients for Cardiac Index and Clinical Variables

### Cardiac Biomarker Data

Using a standard cTnI cutoff value of 0.1 ng/mL, 48% of patients overall (*n* = 50), 42% of SM patients (*n* = 18), and 53% of SMA patients (*n* = 32) had elevated levels of cTnI at admission (20, 22, 29). Using a BNP cutoff value of 100 pg/mL, 19% of all participants (*n* = 20), 7% of SM patients (*n* = 3), and 28% of SMA patients (*n* = 17) had elevated levels of BNP at T0 (19, 23, 24, 30–32).

We examined clinical and laboratory correlates with cardiac biomarkers and found no correlation with age, MUAC, respiratory or HR, pulse oximetry, blood pressure, lactate, hemoglobin, CI, or EF. cTnI and BNP levels correlated well (*r* = 0.850; *p* < 0.001) (**Supplemental Table 1**, Supplemental Digital Content 1, http://links.lww.com/PCC/A580).

## DISCUSSION

In this study, we assessed cardiac function using echocardiography and cardiac biomarkers in children presenting with severe *P. falciparum* malaria. The data represent the largest sample size to date describing cardiac physiology in SM in children. Our echocardiographic data demonstrate that most children (96.2%) with severe *P. falciparum* malaria have normal EF despite some elevation of the cardiac biomarkers. In addition, we demonstrated that CI was within normal range for children with SM but moderately elevated in cases of SMA at admission but then normalized following treatment (whole blood transfusion and antimalarials). Given that the primary difference in CI was a function of the VTI, rather than HR, which was actually higher in SM, we conclude that this observed difference in CI for patients with SMA is a function of increased SV in children with SMA. Children with SMA had significantly higher median lactate, increased respiratory rate, and lower MAP, thus lower systemic vascular resistance. Thus, decreased afterload also may account for the higher SV in the SMA compared with SM group.

Children in both the SM and SMA groups were matched for age and gender; however, there was a small difference in median MUAC, 15.8 versus 13.5 cm. Eleven of 104 children were in the “orange” zone (moderate acute malnutrition), and the remainder were in the “green” zone (normal range) for MUAC. No patients were severely malnourished. Prior data have demonstrated a preserved CI in malnutrition when adjusted for TBSA (33). As such, we believe that nutritional status did not significantly affect our results.

We found mild elevation (> 100 pg/mL) of BNP levels in 19% of patients (*n* = 20) with SM. No patients had markedly elevated levels of BNP (range, 22–386 pg/mL). BNP values in our cohort were above the reference range for healthy children and were consistent with levels seen in critical illness (19, 21, 23, 24, 30, 32, 34, 35). Commonly used cutoff values for BNP in children with cardiac dysfunction are variable; however, the elevation in BNP seen in our cohort was below than those commonly used to identify LV dysfunction (25, 26, 36, 37). Given that very few children had echocardiographic evidence of depressed EF, and the fact that BNP levels were below than those commonly used to identify LV dysfunction, we conclude that LV failure is not a significant contributor to the observed mortality in children with SM.

The frequency of cTnI elevation in our cohort was notable, 48% having elevated levels of cTnI. The level of troponin elevation was similar to that of reported values in pediatric patients with severe sepsis (29, 38, 39). In our patients, there was no correlation with either cTnI or BNP and any clinical or echocardiographic variables. We suggest that BNP and cTnI elevations, well described in both adults and children with severe sepsis, are likely due to myocardial stress in the context of critical illness and severe anemia rather than indices of a failing myocardium (21, 29, 38). As a significant proportion of children (48%) had elevated cTnI levels and only 3.8% had evidence of depressed EF, we conclude that cardiac dysfunction and myocardial injury (as evidenced by elevated troponin) are not related in a direct causal pathway in pediatric patients with SM and instead reflect a state of hypoperfusion secondary to shock and anemia. In the context of SM, cTnI elevation may also be due to RBC sequestration in the coronary microvasculature.

Reference values of CI in healthy children indicate a median of 4.5 L/min/m^2^ (28). Median values for CI in our cohort were slightly elevated in children with SM and significantly elevated in children with SMA at T0 (Fig. 1). At T1, following resuscitation, CI was close to reference range in both groups. Previous studies describing cardiac function in SM have shown discordant results. Yacoub et al (11) demonstrated mild depression in cardiac function that was more pronounced in children with acidosis. In this study, SV improved after fluid bolus and normalized at discharge; however, sample size was small, *n* equals to 30. Murphy et al (12) found no qualitative evidence of myocardial dysfunction in children with and without severe anemia, and Mocumbi et al (40) also reported normal qualitative ventricular function in children with SM. Sample sizes in both of these studies were small, 26 and 45, and both studies used qualitative measures. Our data suggest that cardiac output in severe *P. falciparum* with SMA is increased and is a function in increased SV with normal to high EF. This increase in cardiac output normalized following transfusion and antimalarial therapy and is consistent with an appropriate response to anemia and shock.

In pediatric bacterial sepsis, distinct hemodynamic patterns characterized as either warm or cold shock have been observed with the type of bacterial pathogen determining the nature of cardiac dysfunction (41). In pediatric sepsis with refractory shock, cardiac function is often depressed and correlates with poor outcomes (42, 43). Although inflammatory mediators and cytokine release, with resultant vasodilatory shock, with or without “myocardial stunning” is well described in bacterial sepsis, the role of proinflammatory cytokines in SM are not well defined (41, 42, 44–48). As previous studies in pediatric sepsis have found increased survival when CI was between 3.3 and 6 L/min/m^2^, the ranged deemed to be “normal,” we conclude that children with SM have preserved cardiac function. Only two children in our cohort had a CI less than 3.3 L/min/m^2^ (3.0 and 3.1 L/min/m^2^). Both of these children had SMA and recovered without complication.

Our data provide the first comprehensive clinical, laboratory, and echocardiographic description of cardiac physiology in SM. Our data demonstrated that children with SMA had significantly elevated CI, which normalized following blood transfusion and antimalarial therapy. We also detected a moderate correlation between [hemoglobin] and CI, such that as [hemoglobin] decreases, CI increases. We suggest that the primary physiologic driver of the observed increase in CI is likely that of severe anemia and is a normal adaptive response to anemia and shock with an increase in SV.

As we were not able to definitively identify children with concomitant bacterial infection, which is likely, we cannot draw conclusions regarding the contribution of bacterial coinfection. The use of VTI to calculate CI depends on accurate and reproducible measurement of the LVOT. As such, any inaccuracies in the measurement of LVOT may be reflected in the comparison of CI at T0 and T1.

## CONCLUSIONS

Children with SM have preserved myocardial systolic function. CI is elevated in SMA and correlates inversely with [hemoglobin]. cTnI is elevated in a significant proportion of children with SM but does not correlate with myocardial dysfunction. The physiologic mechanism for the increase in CI in SMA is increased SV and is an appropriate physiologic response. Following blood transfusion and antimalarial therapy, CI in SMA returns to baseline.

## ACKNOWLEDGMENTS

We would like to acknowledge the support of Philips Medical in providing loaned ultrasound equipment for this study. We would also like to thank the clinical nursing team at Mbale Regional Referral Hospital for their diligence and dedication.

## Supplementary Material
