# Supplementary material for: Assessment of Myocardial Function and Injury by Echocardiography and Cardiac Biomarkers in African Children With Severe Plasmodium falciparum Malaria*
Source: Pediatr Crit Care Med. 2018 Mar 2;19(3):179–85. doi: 10.1097/PCC.0000000000001411 (PMC5835359; doi:10.1097/PCC.0000000000001411)
Supplement: Supplementary file 1 [file pcc-19-179-s001.docx]

**Supplemental Table 1: Correlation of laboratory and clinical features with cardiac biomarkers**

|  | Pearson Coefficient | | Pearson Coefficient | |
| --- | --- | --- | --- | --- |
|  | BNP | p-value | cTnI | p-value |
| **Age** | 0.007 | 0.948 | 0.161 | 0.103 |
| **MUAC** | -0.119 | 0.955 | -0.013 | 0.903 |
| **Heart rate** | -0.13 | 0.216 | -0.129 | 0.218 |
| **Respiratory rate** | 0.068 | 0.491 | 0.005 | 0.958 |
| **SpO_2_** | -0.059 | 0.555 | 0.044 | 0.656 |
| **MAP** | -0.009 | 0.926 | -0.077 | 0.451 |
| **Hemoglobin** | -0.117 | 0.236 | -0.098 | 0.321 |
| **Lactate** | 0.065 | 0.516 | -.039 | 0.697 |
| **Ejection Fraction** | -0.153 | 0.121 | -0.145 | 0.142 |
| **Cardiac Index** | 0.199 | 0.231 | 0.091 | 0.357 |
| **BNP** |  |  | 0.850^*^ | <0.001 |
| **cTnI** | 0.850^*^ | <0.001 |  |  |
| **Outcome** | -0.057 | 0.567 | 0.000 | 0.999 |

BNP Brain-Naturetic-Peptide

cTnI Cardiac Troponin I

MUAC mid-upper arm circumference

MAP mean arterial pressure
